# Depressive symptoms are not associated with inflammation in younger and older adults in the Philippines

**DOI:** 10.1093/emph/eos004

**Published:** 2012-12-24

**Authors:** Thomas W. McDade, Judith B. Borja, Linda S. Adair, Christopher Kuzawa

**Affiliations:** ^1^Department of Anthropology, Northwestern University, Evanston, IL 60208, USA; ^2^Cells to Society (C2S): The Center on Social Disparities and Health, Institute for Policy Research, Northwestern University, Evanston, IL 60208, USA; ^3^USC-Office of Population Studies Foundation, Inc., University of San Carlos, Cebu City, Philippines; ^4^Carolina Population Center and Department of Nutrition, University of North Carolina, Chapel Hill, NC, USA.

**Keywords:** psychoneuroimmunology, infectious disease, cardiovascular disease, developmental origins of adult disease, human ecological immunology

## Abstract

Depression is positively associated with chronic inflammation in industrialized settings with low burdens of infectious disease, but the pattern of association in environments with higher levels of microbial exposure is not known. We measured C-reactive protein (CRP) and interleukin 6 (IL6) in community-based samples of young adults (20–22 years) and older women (35–69 years) in the Philippines. Concentrations of CRP and IL6 were low, and bivariate and multivariate regression analyses indicated no associations between depressive symptoms and inflammation in either sample. Results are interpreted in light of prior research indicating that higher levels of microbial exposure in infancy have lasting effects on the regulation of inflammation, and may prevent the emergence of a relationship between depression and inflammation in adulthood.

## Abstract (precis)

Depression is not associated with inflammation among adults in the Philippines, in contrast to prior research in the US. These results suggest that higher levels of microbial exposure in the Philippines may promote the development of immuno-regulatory pathways that prevent the emergence of a relationship between depression and inflammation.



## INTRODUCTION

Prior research has documented a robust dose–response relationship between depression and inflammation in clinic- and community-based samples, with evidence in support of bidirectional causal pathways [1]. Circulating mediators of inflammation, including C-reactive protein (CRP) and interleukin 6 (IL6), have emerged as significant predictors of cardiovascular disease risk [2], pointing to inflammation as a potentially important mediator of the association between depressive symptoms and cardiovascular disease [3].

A limitation of prior research is that it has been conducted almost exclusively in high-income, industrialized settings with low levels of infectious disease. Inflammation plays a central role in innate immune defenses against infection, and the human immune system evolved in environments where the intensity and diversity of microbial exposure was substantially higher [4]. Exposures to infectious microbes during sensitive periods of immune development play important roles in establishing pathways involved in the regulation of inflammation, and divergent patterns of microbial exposure across ecological settings may have significant implications for the impact of depression on inflammation, and vice versa.

We investigate the relationship between depressive symptoms and two biomarkers of inflammation—CRP and IL6—among two cohorts of adults in the Philippines. When the young adults in the study were born in the early 1980s, approximately half the homes had electricity, more than three quarters collected water from an open source, less than half used a flush toilet and more than half had animals roaming under, around or in the house. The level of exposure to infectious microbes was relatively high, and episodes of diarrhea were frequent. Infectious diseases continue to account for significant burdens of morbidity and mortality in the Philippines, even as rates of chronic degenerative disease are on the rise [5], providing a distinct ecological setting for comparison with prior research on depression and inflammation.

## METHODOLOGY

### Participants and data collection

The Cebu Longitudinal Health and Nutrition Survey began in 1983 with the recruitment of 3327 pregnant women in Cebu City, Philippines. For this analysis, we draw on parallel sets of data collected in 2005 for both cohorts: the offspring, now young adults (average age = 20.9 years, range = 20–22 years), and their mothers, now older women (average age = 48.4, range = 35–69 years). Complete data were available for 1604 non-pregnant young adults and 1881 older women. The interview and blood collection aspects of the 2005 survey were implemented in different phases. For young adults, the median interval between interview and blood collection was 55 days. For older women, the interval was 51 days. All data were collected under conditions of informed consent with institutional review board approval from the University of North Carolina, Chapel Hill. Additional information on study design, data collection protocols and reasons for sample attrition has been published previously [5].

### Analysis of CRP and IL6

Venipuncture blood samples were collected into EDTA-coated vacutainer tubes in participants’ homes in the morning after an overnight fast. Samples were kept in coolers on ice packs during transport for no more than 2 h and were then centrifuged at the University of San Carlos to separate plasma prior to freezing at −70°C. Samples were express shipped to Northwestern University on dry ice and stored frozen at −80°C until analysis. CRP concentrations were determined in young adults and older women using a high sensitivity immunoturbidimetric method (Synchron LX20, lower detection limit: 0.1 mg/L). Concentrations of IL6 were determined in samples from young adults only, using a high sensitivity immunoassay protocol (HSCYTO-60SK, Millipore, Billerica, MA, USA) on the Luminex platform (Luminex Corporation, Austin, TX, USA) (lower detection limit: 0.10 pg/ml).

### Measurement of depressive symptoms

Depressive symptoms were assessed in 2005 using questions adapted from the Center for Epidemiologic Studies-Depression Scale (CES-D) and previously applied in the Philippines [6]. Respondents were asked to rate how often in the preceding 4 weeks they had experienced feelings or problems such as difficulty sleeping or eating, feeling lonely, feeling worried, people were unfriendly, people disliked you, feelings of worthlessness and suicidal thoughts. Positively framed items included feeling happy, hopeful about the future and enjoying daily life. Ratings on a three-point scale ranged from ‘none of the time’ (1), ‘occasionally’ (2), to ‘most or all of the time’ (3). Scores were summed across all 15 questions, following reverse scoring of positive items, to generate an index of depressive symptoms. Cronbach’s α was 0.742 for young adults and 0.760 for older women, indicating a high degree of reliability in both samples.

### Data analysis

We first analyzed the unadjusted association between CRP and depressive symptoms in young adults and older women and between IL6 and depressive symptoms in young adults. We then evaluated regression models adjusting for covariates which may confound associations between depressive symptoms and inflammation, including age, gender, waist circumference, skinfold thickness, household sanitation, household assets, household income, education, smoking, oral contraceptive use and use of anti-inflammatory medication. In addition, for the young adults we included variables representing exposures in infancy: birth weight, season of birth, frequency of diarrhea and intensity of exposure to animal feces [5].

We applied Tobit regression models for censored data to account for the substantial proportion of values below the detection limits of the CRP and IL6 assays. In addition, we replicated all models with logistic regression using CRP >3 mg/l (corresponding to increased cardiovascular risk) [2] and IL6 >2.1 pg/ml (corresponding to the top tertile of the sample distribution) as outcomes. The depressive symptom index was included in all models as a continuous variable, but we also considered a categorical version based on quintiles of the distribution in order to detect non-linear or threshold associations.

Concentrations of CRP and IL6 increase as part of the acute phase response to infectious disease, and under this circumstance do not represent baseline levels of chronic inflammatory activity that are associated with depressive symptoms and risk for chronic disease [2, 3]. Therefore, *N* = 228 young adults and *N* = 315 older women reporting symptoms of infectious disease at the time of blood collection were removed from the analyses. Excluded young adults had higher median concentrations of CRP (0.6 vs. 0.2 mg/l) and IL6 (1.8 vs. 0.9 pg/ml), as expected, but did not differ significantly in depressive symptom score, waist circumference or household income. Excluded older women were similarly comparable to those remaining in the sample, but had higher median CRP (1.9 vs. 0.8 mg/l).

## RESULTS

The median CRP concentration for the entire sample of young adults was 0.2 mg/l (interquartile range: 0.1–0.9 mg/L) and did not differ by gender. The median IL6 was 1.0 pg/ml (interquartile range: 0.3–3.3 pg/ml) and also did not differ by gender. As described previously [7, 8], concentrations of CRP and IL6 are low in this sample in comparison with prior research in the USA. The mean depressive symptom score was 21.8 (SD = 3.7), with females having significantly higher scores than males (22.7 vs. 21.0, *P* < 0.001). Two measures of socioeconomic status (household assets: Pearson’s *R* = −0.10, *P* < 0.001; household income: *R* = −0.05, p = 0.07) were negatively associated with depressive symptoms.

**Figure 1.**
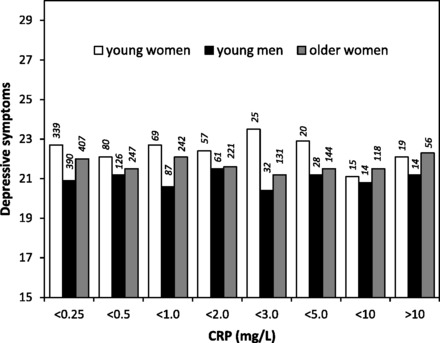
Depressive symptom score in relation to concentrations of CRP among young women and men, and older women, in the Philippines. The number of participants in each group is indicated above the bar.

Descriptive bivariate analyses indicated no association between depressive symptoms and CRP concentration in young adults (Fig. 1). The absence of significant association was confirmed in a fully adjusted Tobit regression model including potentially confounding variables (*β* = 0.003, SE = 0.008, *P* = 0.7). A logistic regression model predicting the likelihood of CRP >3 mg/l also yielded a non-significant association with depressive symptoms [odds ratio (OR) = 0.99, SE = 0.03, *P* = 0.8). The mean depressive symptom score was 21.6 (SD = 3.4) for individuals with CRP >3 mg/l, compared with 21.7 (SD = 3.7) for those with CRP <3 mg/l.

**Figure 2.**
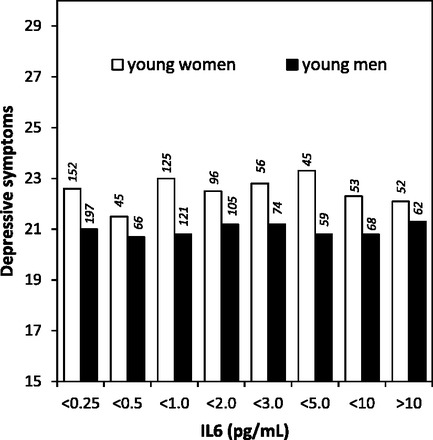
Depressive symptom score in relation to concentrations of IL6 among young women and men in the Philippines. The number of participants in each group is indicated above the bar.

Similarly, depressive symptoms were not associated with IL6 in young adults (Fig. 2). In fully adjusted Tobit and logistic regression models, depressive symptoms did not approach significance as predictors of logIL6 (*β* = −0.007, SE = 0.017, *P* = 0.7) or high IL6 (OR = 1.00, SE = 0.02, *P* = 0.9), respectively. The mean depressive symptom score was 21.7 (SD = 3.8) for individuals with high IL6 and 21.7 (SD = 3.6) for those with lower IL6. We found no evidence for gender differences or non-linearity in the associations between depressive symptoms and IL6 or CRP.

Among the cohort of older women, depressive symptoms were also not associated with CRP (Fig. 1). The median CRP was 0.8 mg/l (interquartile range: 0.2–2.4 mg/l). The mean depressive symptom score was 21.8 (SD = 3.8), and depressive symptoms were negatively correlated with household assets (*R* = −0.18, *P* < 0.001) and household income (*R* = −0.18, *P* < 0.001). Depressive symptoms did not approach significance as predictors of logCRP or CRP >3 mg/l in fully adjusted Tobit regression (*β* = −0.003, SE = 0.005, *P* = 0.6) or logistic regression models (OR = 1.00, SE = 0.02, *P* = 0.9), respectively. The mean depressive symptom score was 21.7 (SD = 3.8) for individuals with CRP >3 mg/l and 21.8 (SD = 3.8) for older women with lower CRP.

Analyses were repeated in models with all participants, including those reporting symptoms of infection at the time of blood collection. The results were very similar. In addition, we re-ran our models with the subset of participants for whom the interval between blood collection and the assessment of depressive symptoms was <30 days. The pattern of results was also similar, with no evidence of positive associations between depressive symptoms and CRP or IL6.

## DISCUSSION

We find no evidence of significant associations between depressive symptoms and two measures of inflammation in large community-based samples of young and older adults in the Philippines. There are at least three possible explanations for this pattern of results, which diverges from prior research documenting positive relationships between depression and inflammation in studies conducted in industrialized settings like the USA [1]. First, it is possible that we did not implement a valid measure of depressive symptoms in the Philippines. However, the index has been used previously in our sample of young adults, where scores were positively associated with witnessing parental domestic violence [6]. In addition, in the analyses above, the index has good internal reliability, a substantial range of variation and scores that correlate with gender and socioeconomic status in expected ways. Nonetheless, prior research has documented the strongest, most consistent associations between depression and inflammation in samples of clinically depressed patients [1], and it is possible that the range of depressive symptoms in our community-based sample is not sufficient to capture an association.

Second, the study may rely on inadequate measures of chronic inflammation, particularly because we assessed CRP and IL6 at only one point in time, and many observations were removed due to the presence of infectious symptoms at the time of blood collection. However, similar designs have been used in many studies demonstrating associations between depression and inflammation, and previously we have shown that CRP, IL6 and measures of adiposity in our sample are positively associated in ways that are consistent with prior research [7, 8]. But our sample is distinct in having exceptionally low concentrations of CRP and IL6 in comparison with prior research. Although we implemented relatively standard, widely applied procedures for the collection and analysis of blood samples in this study, it is possible that issues related to laboratory measurement, sample quality or study design contributed to low concentrations of CRP or IL6 and prevented detection of significant associations with depressive symptoms.

Third, it may be that the low levels of inflammation in our sample and the lack of association between inflammation and symptoms of depression trace back to developmental effects of early environments on the regulation of inflammation. Recently, we have shown that levels of chronic inflammation are lower in environments characterized by a higher prevalence of infectious disease [5, 9], consistent with a broader body of research indicating that microbial exposures during sensitive periods of immune development play important roles in establishing immuno-regulatory networks [4]. In the absence of such exposures, effective anti-inflammatory signaling in particular may be lacking, thereby allowing a positive association between inflammation and depression to emerge. Consistent with this interpretation, Raison *et al.* [10] have recently proposed that reduced contact with tolerogenic microorganisms in high-income industrialized nations is contributing to growing rates of major depressive disorder. In the Philippines, relatively high levels of exposure may have promoted the development of a more tightly regulated inflammatory phenotype that forestalls a causal connection between depression and inflammation. Although these ideas are largely speculative at this point, our hope is that the findings in this study will catalyze additional research in diverse ecological settings that generates insights into the complex associations among depression, inflammation and disease.

## FUNDING

Funding for this study was provided by grants from the National Institutes of Health (RO1HL085144; 5RO1TW05596), including the Fogarty International Center (5RO3TW008133); biomarker data collection was supported by pilot funds from the Interdisciplinary Obesity Center (RR20649) and the Center for Environmental Health and Susceptibility (ES10126; project 7-2004-E).

**Conflict of interest**: none declared.
